# Comparison between video-assisted thoracoscopic lung cancer resection and robot-assisted lung cancer resection: Protocol for a systematic review and meta-analysis

**DOI:** 10.1097/MD.0000000000014790

**Published:** 2019-03-15

**Authors:** Tianci Chai, Yuhan Lin, Zhimin Shen, Sui Chen, Zhenyang Zhang, Wenwei Lin, Peipei Zhang, Mingqiang Kang, Jiangbo Lin

**Affiliations:** a Department of Thoracic Surgery, Fujian Medical University Union Hospital; b The Graduate School of Fujian Medical University; c School of Stomatology, Fujian Medical University, Fuzhou, China.

**Keywords:** lung cancer resection, robot-assisted surgery, video-assisted thoracoscopic surgery

## Abstract

**Background:**

Video-assisted thoracoscopic surgery (VATS) has developed rapidly and a variety of feasible technical methods have been formed. VATS is the main way of lung cancer resection nowadays with minor surgical incision and less bleeding. Robot-assisted thoracic surgery (RATS) is a revolution in surgical procedures and robotic pulmonary resection has been put to use by an increasing quantity of hospitals around the world. However, the widespread adoption of robot-assisted lung cancer resection is controversial. We aimed to evaluate quality metrics of these 2 different approaches of operation by this review and meta-analysis.

**Methods and Analysis:**

We will search Medline, Embase, Pubmed, Google Scholar, and the Cochrane Central Register of Controlled Trials for related literature published in any language before February 28, 2019. Propensity score matched comparative studies, prospective cohort studies; randomized controlled trials (RCTs) will be included. If sufficient data are available, we will perform subgroup analysis in different operative types of lung cancer resection.

**Results:**

The results of this study will be published in a peer-reviewed journal.

**Conclusion:**

To our knowledge, this will be the first time to use meta-analysis to assess quality metrics of video-assisted thoracoscopic lung cancer resection and robot-assisted lung cancer resection. The results of this study will provide more proofs for researchers, clinicians, and patients with lung cancer to choose a suitable surgical procedure. There is not enough high-quality evidence of RCTs to be included, due to the characteristics of interventions. We will try to include some non-randomized controlled trials, small sample trails. Although our team has experience in carrying out a systematic review and meta-analysis, there may be high heterogeneity and low reliability of evidence, which is the limitation of this study.

## 1 Introduction

Lung cancer is the second frequent cancer and the leading common cause of cancer death among men worldwide in 2015.^[1]^ The incidence of lung cancer in women has increased significantly in recent years.^[2]^ Nowadays, surgical resection is one of the main treatments for lung cancer which brings potential healing opportunities to these patients.

In recent years, the techniques of video-assisted thoracoscopic lung cancer resection have made great progress and become the main surgical procedure for lung cancer resection worldwide^[3,4]^ which possesses the advantages of less postoperative pain,^[5,6]^ shorter hospital stay, fewer complications, and lower mortality rates compared with open techniques.^[7–12]^ With advances in science and technology, robotic surgery systems have been invented to extend the surgeon's eyes and hands through computers, which have the advantage of traditional minimally invasive surgery, The utilization rate of robotic surgery technology is rising year by year.^[13,14]^ Compared with video-assisted thoracoscopic surgery (VATS), robotic lung resection has shorter hospital days, more radical lymph node dissection, fewer bleeding, and more accurate surgical incisions.^[15–18]^

However many clinical studies have shown that there is no difference between the time of surgery, nodal harvests, blood loss, and 30-day mortality rate.^[19–21]^ Moreover, the costs of robot-assisted lung cancer resection are higher than video-assisted thoracoscopic lung cancer resection,^[22,23]^ making the promotion of robotic surgery controversial. Emerging technologies need time to improve and evolve so that they can be widely adopted because some high-quality (Hi-Q) studies have been published, in recent years we will conduct meta-analysis and a systematic review of these high quality evidence to evaluate the difference in the quality metrics of these 2 approaches of operation. If sufficient data are available, we will perform subgroup analysis in different operative types of lung cancer resection.

## 2 Objective

A meta-analysis and systematic review will be conducted to estimate the effects of robot-assisted thoracic surgery (RATS) versus VATS for patients with resectable lung cancer.

## 3 Methods

This protocol is conducted adhere to the Preferred Reporting Items for Systematic Review and Meta-Analysis Protocols (PRISMA-P) statement.^[24]^ The results of this systematic review and meta-analysis will be reported according to the Preferred Reporting Items for Systematic Reviews and Meta-Analyse (PRISMA) guidelines.^[25]^ This protocol has been registered in the PROSPERO network (registration number: CRD42018111864).

### 3.1 Eligibility criteria

#### 3.1.1 Types of studies

We will search and include studies that about comparisons between video-assisted thoracoscopic lung cancer resection and robot-assisted lung cancer resection. Propensity score matched comparative studies, prospective cohort studies; randomized controlled trials (RCTs) will be included, without publication type and language restrictions.

#### 3.1.2 Types of participants

The participants included will be adults diagnosed with lung cancer cytologically or histologically confirmed and who were treated with surgical resection of lung cancer. No restrictions regarding race/ethnicity, sex, economic status, and education will be applied.

#### 3.1.3 Types of interventions

All forms of video-assisted thoracoscopic lung cancer resection compared with robot-assisted lung cancer resection for patients with lung cancer.

#### 3.1.4 Types of outcome measures

Comparison of length of hospitalization, complication rate, overall survival, and disease-free survival (DFS) between video-assisted thoracoscopic lung cancer resection and robot-assisted lung cancer resection.

#### 3.1.5 Exclusion criteria

Case reports, case series, review articles, non-peer reviewed articles, animal studies, letters to the editor, editorials, meeting abstracts, commentaries, non-propensity-matched comparative studies, proceedings, and other non-related studies will be excluded.

### 3.2 Information sources

We will search Medline, Embase, Pubmed, Google Scholar, and the Cochrane Central Register of Controlled Trials for related literature published in any language before February 28, 2019.

### 3.3 Search strategy

The relevant keyword or combination subject terms of search will correspond to Medical Subject Heading terms. Before final analysis, the searching will be repeated to identify additional studies meeting the inclusion criteria. The search strategies for PubMed are shown in Table 1.

**Table 1:**
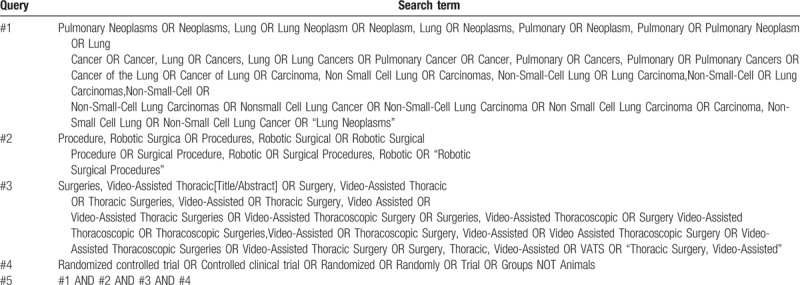
PubMed search strategies.

### 3.4 Data collection and analysis

#### 3.4.1 Study selection

Two reviewers (TCC, YHL) will independently investigate titles and abstracts of all the literature searched and assess whether the studies conform to the inclusion criteria as described in the protocol. The full text of all possible eligible studies will be retrieved and 2 review authors (TCC, YHL) will separately screen the full text to identify the studies to be included. Two reviewers (TCC, JBL) will document the reasons for excluded studies that do not meet the inclusion criteria. Disagreement will be resolved by discussion or, if necessary, in consultation with the third reviewer (ZYZ or WWL). Duplicate literature will be excluded and the multiple reports from the same study will be included in the review of 1 unit. The selection process will be recorded in detail and shown in the PRISMA flow chart.

#### 3.4.2 Data extraction and management

The following data will be abstracted from the included studies: study characteristics (first author, country, publication date, study design, withdrawals, periods of data collection, blanking periods, follow-up duration, total duration of study, et al); characteristics of participants (sex, age, height, weight, smoking and dust exposure, diabetes, hypertension, diagnostic criteria, pathological confirmation, staging of lung cancer according to the TNM classification, etc); intervention characteristics (surgical approach, bleeding, transfusion, duration, thoracotomy conversion, number of lymph nodes retrieved, etc); outcome and other date (length of hospitalization, length of intensive care unit (ICU) stay, complication rate, overall survival, disease-free survival (DFS), etc). All the extracted data will be recorded in a pre-designed excel table and if the relevant data was lost or unclear, we will consult the authors by email before the study are excluded because the data is unavailable.

### 3.5 Assessment of risk of bias

The Cochrane Handbook for Systematic Reviews will be used to evaluate the risk of bias of each study. Two reviewers will assess the risk of bias which based on the following ranges: random sequence generation (selection bias); allocation concealment (selection bias); blinding of participants and personnel (performance bias); blinding of outcome assessment (detection bias); incomplete outcome data (attrition bias); selective outcome reporting (reporting bias); other bias. The assessment results and details will be shown in the risk of bias graph.

### 3.6 Data analysis

We will use Review Manager 5.3 software to synthesize the data extracted. If the data extracted from the included studies are evaluated as highly homogeneous. We will conduct meta-analysis on them for the purpose of obtaining a clinically meaningful result, in order to carry out a standard meta-analysis. Higgins I^2^ statistic and Cochran Q will be used to assess heterogeneity between studies. If the I^2^ or Chi^2^ statistic>50% will be considered to be highly heterogeneous and the data will be analyzed by a random effect model. Otherwise, we will adopt the fixed effect model to analysis the data. Mantel–Haenszel method will be used in the pooling of binary data and the results will be presented in the form of relative risk (RR) about 95% confidence interval (CI) of the dates. The inverse variance method will be applied in pooling of continuous data. The results will be given in the form of standardized mean difference (SMD) with 95% CI.

#### 3.6.1 Subgroup analysis

Subgroup analysis will be conducted to search potential sources of heterogeneity and if sufficient data are available, we will perform subgroup analysis in different operative types of lung cancer resection.

#### 3.6.2 Sensitivity analysis

To determine whether the aggregation results are robust and reliable, sensitivity analysis will be conducted by excluding highly biased studies.

### 3.7 Publication bias

We will use funnel plots and Egger tests to qualitatively analyze publication bias of the studies included. When publication bias does exist, we will use trim and fill method to analyze publication bias in the studies.^[26]^

### 3.8 Evidence evaluation

The overall evidence will be assessed by the Grading of Recommendations, Assessment, Development, and Evaluation (GRADE) approach and the quality of evidence will be assessed as 4 levels—high, moderate, low, and very low ^[27]^

## 4 Discussion

The latest studies show that the incidence of lung cancer is increasing year by year worldwide, which has become the first frequent cancer and the leading common cause of cancer death in human worldwide.^[2]^ Lung cancer resection plays an important role in providing a potentially curable chance for lung cancer patients.

Robot-assisted lung cancer resection has an indisputable technological advantage over traditional video-assisted lung surgery. Robot surgery has better accuracy, comfort, and stereoscopic vision. RATS is a safe and promising alternative to VATS for difficult lobectomy. Early studies have shown that robotic surgery has no advantage versus VATS on postoperative outcomes and the cost of robotic surgery is very high, so the promotion of robotic surgery technology is controversial.

Due to the latest high-quality clinical studies published, we will use the advantages of this high-quality evidence for systematic review and meta-analysis to obtain objective results. This result will provide a reference for clinical decision making. There is still a long way to go before the popularization of robotic surgery. We hope that robotic surgery technology will be further improved and the cost reduced to make a great contribution to human health.

## Author contributions

Jiangbo Lin and Mingqiang Kang is the guarantor of the article. Tianci Chai, Yuhan Lin, and Jiangbo Lin conceived and designed the study. Tianci Chai, Yuhan Lin, Zhimin Shen, and Sui Chen drafted this protocol. Zhenyang Zhang, Wenwei Lin, Peipei Zhang, and Zhimin Shen will perform the search, screening, and extraction. Jiangbo Lin and Mingqiang Kang have strictly reviewed this protocol and approved of publication. Tianci Chai and Yuhan Lin contributed equally to this work.

**Conceptualization:** Tianci Chai, Yuhan Lin, Jiangbo Lin.

**Data curation:** Tianci Chai, Yuhan Lin.

**Formal analysis:** Tianci Chai, Yuhan Lin.

**Funding acquisition:** Yuhan Lin, Mingqiang Kang.

**Investigation:** Tianci Chai, Yuhan Lin, Zhimin Shen, Wenwei Lin, Peipei Zhang.

**Methodology:** Tianci Chai, Zhimin Shen, Wenwei Lin.

**Project administration:** Tianci Chai.

**Resources:** Sui Chen.

**Software:** Yuhan Lin, Zhimin Shen, Sui Chen, Zhenyang Zhang, Wenwei Lin, Peipei Zhang.

**Supervision:** Wenwei Lin, Mingqiang Kang, Jiangbo Lin.

**Validation:** Zhimin Shen, Sui Chen.

**Visualization:** Sui Chen.

**Writing – original draft:** Zhenyang Zhang, Mingqiang Kang, Jiangbo Lin.

**Writing – review & editing:** Mingqiang Kang, Jiangbo Lin.
